# 

**Published:** 1883-12

**Authors:** 


### Pamphlets.

The Medical Record Visiting List, or Physician's Diary, for 1884. New York: Wm. Wood & Co. We hereby return our thanks to this enterprising publishing house for a copy of their unsurpassed Physician's Diary—beautiful in style and most convenient and helpful in arrangement.

The Physician's Visiting List for 1884—39th year of its publication. Philadelphia: P. Blakiston, Son & Co., 1012 Walnut street. Our thanks are due to this well-known firm for a copy of the above work. It contains many admirable features, and is deservedly popular.

The Physician's Memorandum Book. Arranged by Joel A. Miner. Fifth improved edition, with clinical columns and ledger sheets. Ann Arbor, Mich.: Joel A. Miner, publisher. Order by mail. We carry a copy of this excellent book constantly in pocket, and it has never failed us in an emergency.

The increase of insanity in the United States, its causes and sources. By Foster Pratt, M. D., Kalamazoo, Mich. A paper read before the "American Health Association," at Detroit, Mich., Nov. 1883.

